# On Developing Field-Effect-Tunable Nanofluidic Ion Diodes with Bipolar, Induced-Charge Electrokinetics

**DOI:** 10.3390/mi9040179

**Published:** 2018-04-12

**Authors:** Ye Tao, Weiyu Liu, Yukun Ren, Yansu Hu, Guang Li, Guoyun Ma, Qisheng Wu

**Affiliations:** 1 State Key Laboratory of Robotics and System, Harbin Institute of Technology, West Da-zhi Street 92, Harbin 150001, Heilongjiang, China; tarahit@gmail.com; 2 School of Electronics and Control Engineering, Chang’an University, Middle-Section of Nan’er Huan Road, Xi’an 710064, Shaanxi, China; huyansu@chd.edu.cn (Y.H.); GuangLi@chd.edu.cn (G.L.); 201432010615@chd.edu.cn (G.M.); qshwu@chd.edu.cn (Q.W.)

**Keywords:** nanofluidics, induced-charge electrokinetic phenomenon, linear and nonlinear electroosmotic streaming, concentration polarization

## Abstract

We introduce herein the induced-charge electrokinetic phenomenon to nanometer fluidic systems; the design of the nanofluidic ion diode for field-effect ionic current control of the nanometer dimension is developed by enhancing internal ion concentration polarization through electrochemical transport of inhomogeneous inducing-counterions resulting from double gate terminals mounted on top of a thin dielectric layer, which covers the nanochannel connected to microfluidic reservoirs on both sides. A mathematical model based on the fully-coupled Poisson-Nernst-Plank-Navier-Stokes equations is developed to study the feasibility of this structural configuration causing effective ionic current rectification. The effect of various physiochemical and geometrical parameters, such as the native surface charge density on the nanochannel sidewalls, the number of gate electrodes (GE), the gate voltage magnitude, and the solution conductivity, permittivity, and thickness of the dielectric coating, as well as the size and position of the GE pair of opposite gate polarity, on the resulted rectification performance of the presented nanoscale ionic device is numerically analyzed by using a commercial software package, COMSOL Multiphysics (version 5.2). Three types of electrohydrodynamic flow, including electroosmosis of 1st kind, induced-charge electroosmosis, and electroosmosis of 2nd kind that were originated by the Coulomb force within three distinct charge layers coexist in the micro/nanofluidic hybrid network and are shown to simultaneously influence the output current flux in a complex manner. The rectification factor of a contrast between the ‘on’ and ‘off’ working states can even exceed one thousand-fold in the case of choosing a suitable combination of several key parameters. Our demonstration of field-effect-tunable nanofluidic ion diodes of double external gate electrodes proves invaluable for the construction of a flexible electrokinetic platform for ionic current control and may help transform the field of smart, on-chip, integrated circuits.

## 1. Introduction

With the incessant development of nanoscience, interfacial force effects have received increasing attention from the micro/nanofluidic community, due to their benign scaling with larger surface-to-volume ratios in miniaturization systems. Among them, diffuse-charge dynamics next to a Debye screening cloud constitute a significant polarization phenomenon at the sharp material interface between a solid surface and the adjacent saline solution: the native free surface charge chemically adsorbed on the channel walls is always compensated for by a thin electrical double layer (EDL) of mobile counterionic charges in the liquid phase from which coions are forced to leave [1,2,3,4]. Since the formation of the counterionic charged cloud is due to a dynamic equilibrium between electrophoretic transport and the thermal diffusion of ionic species in a normal direction to the phase interface, the extension of the Debye screening layer depends strongly on the background ionic strength and can transform from smaller than 1nm at high ion concentrations to more than 100 nm in low-conductivity working fluids [5,6,7,8,9,10,11,12,13].

Within a nanometer-sized room, the diffuse charge cloud represents a significant section of the entire volume, and therefore the surface conductivity within the thin Debye layer dictates in front of bulk conduction [14,15,16]. For example, nanofluidic ducts with characteristic dimensions on the level of double-layer thickness are capable of possessing unique ion motion characteristics; with an evident EDL extension, the surface charge density naturally developed on the channel sidewalls, resulting in the ionic contents being in a high degree of asymmetry, with a global difference in ion concentrations between cations and anions [17,18,19]. As a result, only counterionic charges are permitted to translate through nanochannels, while the axial transport of coions is inhibited [3]. Accordingly, surface-charge-governed electrokinetics in nanochannels (in which overlap of native double-layers takes place on account of the thin channel width) is quite different from electrokinetic phenomena in microfluidic devices [17,20,21,22,23,24,25,26,27,28]. Most significantly, in the presence of ion-selective motion with respect to the wall charge of opposite polarity, ionic current flux can be controlled within surface-conduction-dominated nanofluidic channels, while it is not possible to realize this in microchannels governed by bulk conductance [29,30,31].

**Figure 1:**
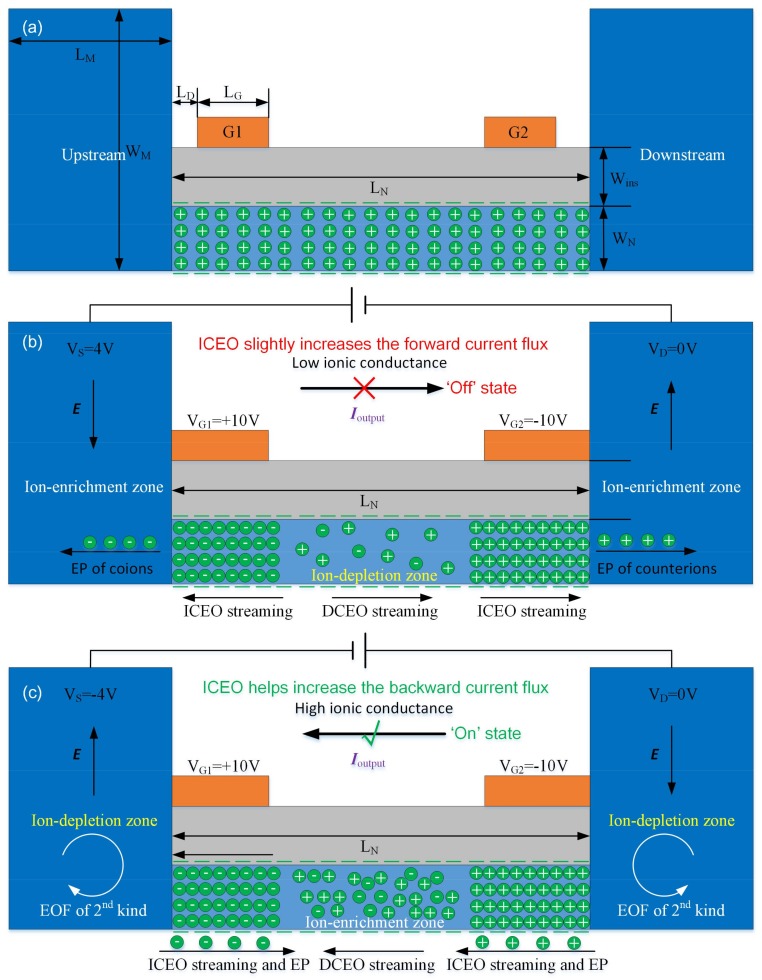
2D Schematics of electrical manipulation strategy for the two distinct working statuses and rectification mechanism of output ionic current *I*_output_ of the presented nanofluidic ion diode with double GEs laid on top of a dielectric coating layer to close the nanochannel of negative wall charge. (**a**) Geometric configuration of the nano-scale device. (**b**,**c**) Under positive V_G1_ and negative V_G2_; (**b**) the output current flux *I*_output_ towards the downstream microchamber is inhibited under a positive value of Vs > 0, in that an ion depletion zone is produced in the central district by the vast transport of induced counterions toward adjacent reservoirs, i.e., the ‘off’ status; and (**c**) *I*_output_ towards the upstream reservoir can be switched on with a negative value of Vs < 0, since stable ion enrichment is induced at the midchannel due to fast inward translation of induced counterions to the central region covered by the physical gap between the double GE.

Due to its superiority at generating ionic current rectification, a diversity of nonlinear ionic circuit elements to date have accomplished the functions of ionic diode [31,32], transistor [33,34], or amplifier [35] by modulating ion concentrations inside the nanofluidic space. However, the concentration modulation area usually occupies a finite section of the nanochannels, which suppresses the potential ionic current rectification in such nanodevices. We have recently established a mathematical model [36] for the field-effect-tunable microfluidic ion diode of a gating ion-exchange medium, in which the ion concentration distribution in the output duct can be effectively adjusted, since the depleted/enriched concentration polarization waves can well propagate in the external duct along both current directions, resulting in a high-flux ion rectification for reverse working statues of the ionic device. On the contrary, to make the device smaller for portable applications, we present herein a simple but useful way to set up the field-effect-tunable nanofluidic ion diode by adjusting the internal rather than the external ion concentration distribution of a central nanochannel connected to two reservoirs on both sides, under the help of bipolar induced-charge electrokinetics [37,38,39,40,41,42,43] from the double external gate terminals of the opposite gate polarities (Figure 1). Under the action of an imposed axial electric field between the source (S) and drain (D) terminals, the ion conductance of the nanofluidic channel, within which EDLs from both channel sidewalls occur to overlap under reasonable liquid conductivities, can be altered by causing either ion depletion or ion enrichment at the central section (midchannel) with the vast transport of induced counterions under the covering area of two gate terminals (G_1_ and G_2_). By reshaping buffer ion concentrations at the nanometer dimension, our simulation analysis using a commercial software package, COMSOL Multiphysics (version 5.2, COMSOL Inc., Stockholm, Sweden), indicates the nanofluidic ion diode has great potential to achieve highly-efficient ionic current rectification in small-scale ion circuit platforms.

## 2. Materials and Methods

### 2.1. Basic Device Structure and Operation Principle

Basic geometric structure of our nano-scale ionic device is one nanochannel of L_N_ = 1 μm in length and H_N_ = 10 nm in height, bridging two micro-scale reservoirs of L_M_ = 1 μm in length and H_M_ = 1 μm in height on both sides. A thin layer of dielectric coating is in direct contact with the top wall of nanochannel and covers its whole surface area, having a thickness of H_ins_ = 100 nm. Single or double thin gate (G) terminals of identical length L_G_ = 100 nm are deposited on both sides on top of the dielectric layer, in order to achieve field-effect-control on transport of charge carriers inside the thin duct. In this way, any motion of ionic species from the source (S) to drain (D) terminals under an imposed DC electric field occurs perpetually through the charged nanochannel. Accordingly, the fluid path across S–D terminals function as the output fluidic channel of the micro/nanofluidic hybrid system. The usage of double-side GEs makes ion conductivity at the midchannel stipulate the global ionic current flux flowing throughout the fluidic device.

Both the top and bottom nanochannel sidewalls are supposed to possess a uniform distribution of fixed free surface charge density of a negative sign, e.g., *σ_free_* = −0.0001 C/m^2^, from a spontaneous physicochemical polarization process independent of the externally applied voltages, resulting in a native diffuse double layer full of cations, as shown in Figure 1a. Apart from the native positively-charged double layer, the two ideally polarizable GEs positioned externally on top of the insulation layer can give rise to additional induced counterions with respect to the specific gate polarity under their covering area inside the nanochannel due to the action of induced-charge electrokinetic phenomena (ICEK), i.e., anions are induced under G1 of a positive gate voltage V_G1_ = 10 V, and additional cations are induced under G2 of a negative gate polarity V_G2_ = −10 V. Besides, since native and induced electrical double layers (EDL) can actively superimpose on one another, the positive volumetric free charge inside the Debye layer under G2 is always more than the negative charge carriers under G1 (Figure 1b,c).

Under this situation and with the D terminal being constantly grounded (Vd ≡ 0 V), for a positive source voltage, e.g., V_S_ = 4 V, imposed at the S terminal in the left micro-scale reservoir, the rightward background DC electric field across the nanochannel causes very fast transport of a large amount of induced counterionic charges under the covering area of GEs into adjacent microchambers due to mismatch of charge carriers at the micro/nanofluidic interface. This gives rise to an ion-depletion zone in the middle section of the nanochannel, that is, the ion conductance toward the downstream outlet port is greatly lowered, and the forward ionic current flux is switched off under positive Vs, i.e., the ‘off’ working state (Figure 1b). Simultaneously, the convection current due to charge motion in the flow field of induced-charge electroosmosis (ICEO) tends to increase the forward ionic current to a slight degree, which unexpectedly lowers the rectification performance.

On the contrary, for a negative source voltage, e.g., V_S_ = −4 V, lower than the grounding voltage of D terminal, the applied electric field reverses in direction and points from the right to left reservoir. This causes the induced counterions under the covering region of GEs to move into the midchannel due to mismatch of ionic conductance at the interface between covering and non-covering areas of GEs, resulting in ion enrichment at the center. Accordingly, the ionic conductivity toward the source terminal is enhanced to great extent, and the backward electric current flow is well switched on under negative Vs, i.e., the ‘on’ working state (Figure 1c). At the same time, nonlinear electroosmotic streaming inside the nanochannel has a propensity to increase the backward current flux, which helps raise the rectification factor. Meanwhile, it is noteworthy that although an abundance of cations and anions are present at the center of nanochannel, electrolyte charge carriers are concurrently depleted in the reservoirs on both sides to guarantee mass conservation on the global scale. Consequently, electro-convective vortex flow due to electroosmosis of 2nd may appear in the microchambers at a sufficiently large Dukhin number, which describes the competition between non-uniform surface conduction within the thin double layer and Ohmic current injection from the fluid bulk. 2nd electroosmotic flow (EOF) usually causes electrohydrodynamic (EHD) instability in depleted concentration-polarization zone and makes the ionic charge constantly redistribute via charge convection in a fluid flow; this may impose a negative effect on the ion conductance in the backward ‘on’ state. By controlling buffer ion concentrations in a stable manner inside nanoscale space, the nanofluidic ionic circuit platform with double external gate terminals provides useful guidelines for the design of smart integrated circuits in modern micro total analytical system.

### 2.2. Assumption and Approximations Used in Mathematical Analysis

According to electrical manipulation principles of the nanofluidic ionic device, three different kinds of electro-convective streaming, including 1st EOF, ICEO, and 2nd EOF, as caused by the Coulomb force within the native diffuse double layer, the induced Debye screening cloud, and the extended space charge region in ion-depletion zone, respectively, coexist in the functional ionic circuit platform. So, it is imperative to calculate the rectification performance by quantitative mathematical analysis.

For analytical convenience, KCl aqueous solution (a binary symmetric electrolyte of monovalent ions) is employed as the standard working fluid in current work. The nanochannel was regarded as a domain rather than a surface. It is worth mentioning here that our ionic device is easily scalable, as long as an increase in length of the nanochannel is accompanied by a larger size of the dielectric layer and two gate terminals. The length and height of micro-chambers can also be enhanced in an on-demand manner, while larger driving voltages and gate voltages have to be imposed on corresponding terminals to make the nanofluidic device operate effectively at a larger dimension. Distribution of extra-fine grids ~0.2 nm with a maximum growth rate of 1.03 as meshes extending to the channel centerline is imposed at the nanochannel sidewalls to resolve both the native and induced Debye screening clouds within the nanofluidic channel. At the same time, meshes as small as 1 nm have to be specified at both the entrance and exit of nanochannel to recognize the induction of an extended space free charge layer (ESCL) in the microchambers due to non-uniform surface conduction along the overlapped double layers inside the nanochannel.

Although our simulation analysis is in regard to ion delivery in a standard KCl solution, the 10 nm nanochannel height can offer a strongly overlapped double-layer for a broad range of buffer-solution ion strength. Accordingly, the field-effect-tunable nanofluidic ion diode can work with high efficiency, as well for polyelectrolyte in the presence of a variety of charge carriers.

### 2.3. Mathematical Model

According to the continuum mechanics, motion of charge carriers in an electrified nanofluidic device is dictated by the fully-coupled Poisson (Gauss law in conductive medium and dielectric layer) -Nernst-Planck (mass conservation of ionic species) -Navier-Stokes (electroconvection driven by Coulomb force acting on the free charge) equations. For simplicity, we conduct physical descriptions for a binary symmetric electrolyte of 1:1 between cations and anions, such as KCl aqueous solution composed of monovalent charge carriers.

The interrelationship between the molar ion centration distribution and electrostatic potential $\phi_{f}$ in the fluid domain is determined by Gauss law in the conductive liquid medium:

(1) $$\rho_{free}=-\varepsilon_{f}\nabla^{2}\phi_{f}=F\left(C_{+}-C_{-}\right)$$

in which *F* denotes the Faraday constant, and $\varepsilon =80\varepsilon_{0}$ is the real dielectric permittivity of liquid, with $\varepsilon_{0}$ denoting that of the vacuum. The free charge density $\rho_{free}$ is decided by the concentration discrepancy between cations $C_{+}$ and anions $C_{-}$.

On the other hand, there is no free charge in the insulation layer without permanent polarization, and consequently the electrostatic potential $\phi_{ins}$ within the domain of dielectric coating is governed by the Laplace equation:

(2) $$\nabla^{2}\phi_{ins}=0$$

Mass conservation of charge carriers abides by the Nernst-Planck equation:

(3a) $$\frac{\partial C_{+}}{\partial t}+\nabla \cdot \left(-\mu_{+}FC_{+}\nabla \phi_{f}-D_{+}\nabla C_{+}+uC_{+}\right)=0$$

(3b) $$\frac{\partial C_{-}}{\partial t}+\nabla \cdot \left(\mu_{-}FC_{-}\nabla \phi_{f}-D_{-}\nabla C_{-}+uC_{-}\right)=0$$

in which *D*_+_ and *D*_−_ are the diffusion coefficient of positively and negatively-charged ionic species, respectively, with *μ*_+_ and *μ*_−_ representing the mobility in an electric field for corresponding charge carriers. From Einstein relation, *D*_+_/*μ*_+_ = *D*_−_/*μ*_−_ = k_B_TF/*q*, in which k_B_ denotes the Boltzmann constant, T the environmental temperature, and *q* the elementary charge.

Subtracting Equation (3b) from (3a), charge conservation equation for a time-dependent electric field can be acquired:

(4) $$\frac{\partial \rho_{free}}{\partial t}+\nabla \cdot \left(\sigma_{f}E_{f}-F\left(D_{+}\nabla C_{+}-D_{-}\nabla C_{-}\right)+u\rho_{free}\right)=0$$

in which $\sigma =F^{2}(\mu_{+}C_{+}+\mu_{-}C_{-})$ indicates the local ionic conductivity of saline solution. Invoking the Gauss law $\rho_{free}=\nabla \cdot \left(\varepsilon_{f}E_{f}\right)$, the microscopic condition of current continuity has an explicit form:

(5) $$\nabla \cdot \left(\sigma E_{f}+\frac{\partial \varepsilon_{f}E_{f}}{\partial t}-D\nabla \rho_{free}+u\rho {}_{free}\right)=0$$

So, the total current flux can be defined as $J_{total}=\sigma_{f}E_{f}+\frac{\partial \varepsilon_{f}E_{}}{\partial t}-D\nabla \rho_{free}+u\rho_{free}$ in the fluid domain, which is a conserved vector field through the condition of $\nabla \cdot J_{total}=0$. The total current density $J_{total}$ consists of four distinct current components, including Ohmic conduction from charge electro-migration, displacement current due to dielectric polarization, diffusion current from Brownian motion of ions, and convection current due to charge motion in fluid flows, which are indicted by the four terms in sequence on the right hand side of Equation (5), respectively. In this work, since static DC voltage signals are imposed on the source and double gate terminals, the displacement current can be ignored in comparison to other kinds of current fluxes.

Electrokinetic fluid motion in microchambers on both sides, as well as the nanofluidic channel, is governed by the rectified Navier-Stokes equation for incompressible water-based fluids added with a source term of electrical origin:

(6a) $$\rho \frac{\partial u}{\partial t}+\rho \left(u\cdot \nabla \right)u=-\nabla p+\eta \nabla^{2}u+\rho_{free}E$$

(6b) ∇⋅u=0

in which *p* denotes the static hydrodynamic pressure, ***u*** the vector field of fluid motion, ρ the mass density of working fluid, and η the liquid dynamic viscosity. The source term $\rho_{free}E$ represents the electrostatic body force density acting on the free charge within triple charge layers [44], including the native and induced Debye screening clouds inside the nanofluidics, as well as the extended space charge region in depleted zone of reservoirs, which are reflected by the boundary conditions applied at the dielectric layer/fluid interface and gate potentials imposed on both gate terminals (Equations (7) and (9)). Meanwhile, the electrokinetic stress acting on any polarized structural interface is excluded in this work, since all the objects in the nanofluidic device are physically fixed onto the insulating glass substrate in practice.

We do not describe double-layer polarization at charged reservoir walls; however, the electroosmotic fluid motion along the thin duct is much quicker than conventional electroosmosis in microchambers from the view point of momentum conservation. Even so, 2nd EOF due to Coulomb force within the ESCL in ion-depletion zone can still be well reconstructed by our physical model, into which inhomogeneous surface conduction of excessive induced counterions within the nanochannel is incorporated explicitly. The negligence of Debye screening in microchambers avoids the usage of an extra-fine grid distribution adjacent to reservoir walls, making the numerical calculation stay within the limit set by our computer resource. Because the natural diffuse screening cloud is overlooked, ionic charge density within the microchambers comes mainly from the ESCL.

### 2.4. Boundary Conditions

In current work, we establish a 2-D mathematical description for the proposed nanofluidic ion diode with field-effect current control, and carry out multiphysics simulations to comprehend the concentration polarization interface induced inside the nanochannel under the action of two opposite electric fields, ***E***_G1_ and ***E***_G2_, in the vertical direction emitted from the G1 and G2 terminals, respectively, when a DC voltage difference ***E*** is applied in the horizontal direction across the S–D terminals, as shown in Figure 1b,c. Since width of the nanofluidic channel (e.g., 100 nm–2 μm) can be orders of magnitude larger than the nanochannel height (10 nm), it is not possible for the left and right sidewalls of nano-slit to exert a significant influence on the simulation results of various field variables in the lateral direction. For these reasons, it is reasonable to apply 2D approximation for current analysis, in which out-of-plane effects can be dropped safely.

As aforementioned, our mathematical model employs the classical theory of continuum dynamics, since macroscopic equations of electrochemical transport are still viable for typical nanochannel dimension larger than 1 nm. We incorporate the charge convection under the simultaneous impact of 1st EOF (conventional electroosmosis) [45], induced-charge electroosmotic streaming [46,47,48], and the vortex flow field of 2nd EOF [16] (Equation (6a)). 1st EOF is originated by the fixed surface charge defined on the nanochannel sidewalls and Coulomb force within the resulted natural Debye screening cloud (Equation (8)); ICEO is caused by induced polarization of dielectric layer enhanced by the vertical gating electric fields and the electrostatic force acting on the corresponding field-induced diffuse screening cloud, and 2nd EOF is from the Coulomb force within the extended space charge layer due to mismatch of charge carriers at the micro/nanofluidic interfaces. All these electrokinetic flows are reflected by the suitable boundary conditions and source term of electrostatic force inserted in Equation (6a).

Since the quasi-static electric field Equations (1) and (2), ion delivery Equation (3), and electroosmotic flow Equation (6) are mutually coupled, it is actually a challenging mission to tackle this boundary-value problem.

(a) Boundary conditions for potential field

To calculate the electric field in both the liquid domain and insulation layer, Equations (1) and (2) have to be solved at the same time. Since the net conduction current (ohm plus diffusion) vanishes in the normal direction of nanochannel sidewalls from the two distinct phases on both sides (Equation (12)), static free charges can be chemically adsorbed at a solid/liquid interface, which serves as the basis of diffuse-charge dynamics next to insulating charged surfaces. In this way, a distribution of surface charge density can be justifiably designated at the phase interface between the nanochannel and dielectric coating layer, and electrostatic potential has to be continuous at the same time, both of which provide the necessary conjugating conditions at the top wall of the thin duct:

(7a) $$\sigma_{free}=\varepsilon_{ins}\frac{\partial \phi_{ins}}{\partial n}-\varepsilon_{f}\frac{\partial \phi_{f}}{\partial n}$$

(7b) $$\phi_{ins}=\phi_{f}$$

In a similar way, static surface free charge exists on the bottom wall of the nanochannel mounted on an insulating substrate as well:

(8) $$\sigma_{free}=\varepsilon_{f}\frac{\partial \phi_{f}}{\partial n}$$

At various electrode terminals, the electrical potential is determined by external voltage supplies:

(9a) $$\phi =V_{S}\;\mathrm{at}\;S\;\mathrm{terminal}$$

(9b) ϕ=0 at D terminal

(9c) $$\phi =V_{G1}\;\mathrm{at}\;\mathrm{G1}\;\mathrm{terminal}$$

(9d) $$\phi =V_{G2}\;\mathrm{at}\;\mathrm{G2}\;\mathrm{terminal}$$

The total electrical current disappears in the normal direction at other insulating surfaces of zero interfacial charge:

(10) n⋅∇ϕ=0

(b) Boundary conditions for mass transport

As for calculating the distribution of ion concentrations Equation (3), the normal flow flux of cations and anions vanishes on the nanochannel sidewalls, in that electrolyte charge carriers cannot penetrate from the saline solution into adjacent solid phases:

(11a) $$\left(-\mu_{+}FC_{+}\nabla \phi_{f}-D_{+}\nabla C_{+}\right)\cdot n=0$$

(11b) $$\left(\mu_{-}FC_{-}\nabla \phi_{f}-D_{-}\nabla C_{-}\right)\cdot n=0$$

The combination of Equation (11a) and (11b) implies in an implicit manner that the total conduction current is impeded by the sharp material phase:

(12) (σE−D∇ρ)⋅n=0

With the help of Gauss law, a scaling analysis of the boundary condition Equation (12) indicates the counterionic charges mainly exist within a characteristic distance scale of Debye length $\lambda_{D}=\sqrt{D\varepsilon /\sigma }$ away from the charged solid/liquid interface.

Ion concentrations of *C*_+_ = *C*_−_ = *C*_0_ = 1 mM are fixed at the S and D terminals according to actual conditions. Ion electrophoretic mobility *μ*_+_ = *μ*_−_ = 8.21 × 10^−13^ s·mol·kg^−1^ is linearly proportional to the thermal diffusivity of charge carriers *D*_+_ = *D*_−_ = 2 × 10^−9^ m^2^·s^−1^ through Einstein relation within the liquid domain.

(c) Boundary conditions for flow field

The flow behavior of electroosmotic streaming is governed by momentum and mass conservations in Equation (6). The S and D terminals at both ends of the fluidic system are prescribed as open boundaries. No slip-wall boundary is imposed on all other sidewalls.

The governing equations Equations (1)–(3) and (6), which are subjected to given boundary conditions from physical constraints, are in accordance with the unicity theorem. Consequently, current boundary-value problem always has a unique solution at a given set of experimental parameters.

(d) Simulation methodology

As a matter of fact, it can be inferred from the fully-coupled equations presented above that the physical process of electrochemical ion transport in nanochannels interconnected with micro-reservoirs is quite complicated; therefore, it is indispensable to develop a numerical code for providing a physical interpretation of the various electrokinetic phenomena leading to ionic current rectification along the thin duct. Simulation analysis of propagating behavior of the ion concentration polarization (ICP) [49,50] wave in a dual-channel micro/nanofluidic hybrid network has been investigated in our previous work [36], bringing about pathbreaking comprehension on the interaction between ICP and electroosmosis that accounts for the electrokinetic transport of concentration perturbation. In current analysis, however, the situation encountered herein appears to be more complex; the ideally polarizable double G terminals introduce induced-charge electrokinetic phenomena, i.e., counterions of reverse charge polarity are induced under the covering areas of two gate electrodes of opposite voltage polarity, respectively, which makes the net charge density non-uniformly distributed along the nanochannel length direction. This forms the basic physics upon which internal concentration polarization and ionic current rectification in the thin duct are based.

A direct numerical simulation with the fully-coupled mathematical model (Equations (1)–(3) and (6)) demands an extremely fine distribution of meshes (~0.2 nm) within the nanochannel to correctly delineate the diffuse-charge-dynamics-based effects, which is why micrometer reservoirs were often simulated, so as to not exceed the limits of available computer resources. In current analysis, a background ion concentration of *C*_0_ = 1 mM was initially used for both cations and anions, resulting in an Debye length of *λ*_D_ ≈ 10 nm, which implies both the native and induced double layers can be well extended and almost occupy the entire volume of the nanochannel. What is more, extremely detailed grids of 0.1 nm in size were used at the micro/nanofluidic interfaces to resolve the specific structure of extended space charge region in which convective charge transfer becomes very important. In electrokinetic manipulation of the nano-scale ionic device, as a depletion or enrichment region forms at the midchannel, the Debye length would increase or decrease depending on ICP-regulated local ion concentrations, which may be beneficial or detrimental to presenting better calculation accuracy.

We made use of a finite element method (FEM)-based commercial software package, COMSOL Multiphysics (version 5.2), to construct this set of partial differential equations (PDEs) in the context of a scalable 2D nanofluidic device. At the early time, there was a convergence problem. To overcome this rub, we made some changes to the stationary solvers automatically generated by the software, e.g., the scaling method for various dependent variables was artificially changed from automatic (the default setting) to manual with a scale factor of 1.0. In addition, we chose to use the PARDISO solver in priority due to its quicker iteration speed. In this way, numerical convergence of the finite element arithmetic can be ensured.

### 2.5. Scaling Analysis

In this section, we make a scaling analysis of some important variables to judge the approximation condition for the diode behavior to occur. The zeta potential dropped across the diffuse double layer on channel sidewalls is composed of two distinct components in essence. The native zeta potential of Debye screening layer due to the fixed surface charge is given by:

(13a) $$\zeta_{native}\approx \frac{\sigma_{free}\lambda_{D}}{\varepsilon_{f}}$$

Additionally, the induced counterpart due to the polarization of dielectric coating layer under the gating fields is given by:

(13b) $$\zeta_{induced}\approx \frac{V_{G}}{1+\frac{\varepsilon_{f}H_{ins}}{\varepsilon_{ins}\lambda_{D}}}$$

Under the condition of a very low nanochannel height compared to the thickness of dielectric layer (H_N_), the ratio of the two kinds of zeta potentials is given by:

(14) $$\alpha =\frac{\zeta_{induced}}{\zeta_{native}}\approx \frac{\varepsilon_{ins}V_{G}}{H_{ins}\sigma_{free}}$$

To achieve sufficiently strong internal concentration polarization at the midchannel, we must induce a non-uniform distribution of counterionic charges with the two G terminals of opposite voltage polarities, so that the induced zeta potential has to be much larger than the native zeta potential:

(15) $$V_{G}\gg \frac{\sigma_{free}H_{ins}}{\varepsilon_{ins}}$$

using typical parameters in microsystems, e.g., $\sigma_{free}$ = −0.001 C/m^2^, *H_ins_* = 100 nm, $\varepsilon_{ins}=10\varepsilon_{0}$, and $V_{G}\gg$ 2.26 V. As a result, to confirm the threshold gate voltage of double G terminals, beyond which the nanofluidic ion diode has a high performance in ionic current rectification, an appropriate choice is to conduct direct numerical simulation for the fully-coupled boundary-value issue.

Another implicit condition for generating sufficiently large non-uniform surface conductivity is that the Debye length of induced double layer due to polarization of dielectric coating must be no less than the Debye screening length:

(16) $$\lambda_{D}=\sqrt{\frac{D\varepsilon_{f}}{2\mu F^{2}C_{0}}}\geq W_{n}$$

So, the background ion concentration should be no more than a threshold value:

(17) $$C_{0}\leq \frac{{D\varepsilon }_{f}}{2{\mu F}^{2}W_{n}^{2}}\approx 1[\mathrm{mM}]$$

In this sense, as long as ionic circuit platform meets the conditions set by Equations (15) and (17) simultaneously, our nano-scale device configuration can serve as an effective on-chip nanofluidic ion diode. The rectification factor γ is defined as follows:

(18) $$\gamma \left(V_{S}\right)=\left|\frac{I\left({-V}_{S}\right)}{I\left(V_{S}\right)}\right|=\frac{\int_{outlet}J\left({-V}_{S}\right)ds}{\int_{outlet}J\left(V_{S}\right)ds}\times 100\%$$

in which V_S_ and −V_S_ in the brackets indicate the value of corresponding variables for opposite source voltages, I is the electric current at the nanochannel exit, and J is the current density. For instance, the rectification factor γ(V_S_ = 2 V) at V_S_ = 2 V is equal to the absolute value of the division between I(V_S_ = −2 V) and I(V_S_ = 2 V).

## 3. Results and Discussion

### 3.1. Effect of Number of Gate Electrodes on Field-Effect Control of Internal Concentration Polarization

At first, we have to pay attention to the importance of number of G terminals in achieving field-effect ionic current control to see whether it is possible to configure the double G terminals to generate a more stable concentration polarization interface in the middle of the nanochannel than that of single gate electrode. The various physiochemical and geometrical parameters are: L_N_ = 1 μm, W_N_ = 10 nm, L_ins_ = 980 nm, W_ins_ = 100 nm, W_M_ = 1 μm, L_M_ = 1 μm, L_G1_ = L_G2_ = L_G_ = 100 nm, L_D_ = 20 nm, V_G1_ = 10 V, V_G2_ = −10 V, V_S_ = 4 V, *σ_free_* = −0.0001 C/m^2^, and *C*_0_ = 1 mM. The double layer of *λ*_D_ = 10 nm in thickness can be extended right throughout the nanochannel vertical direction.

**Figure 2:**
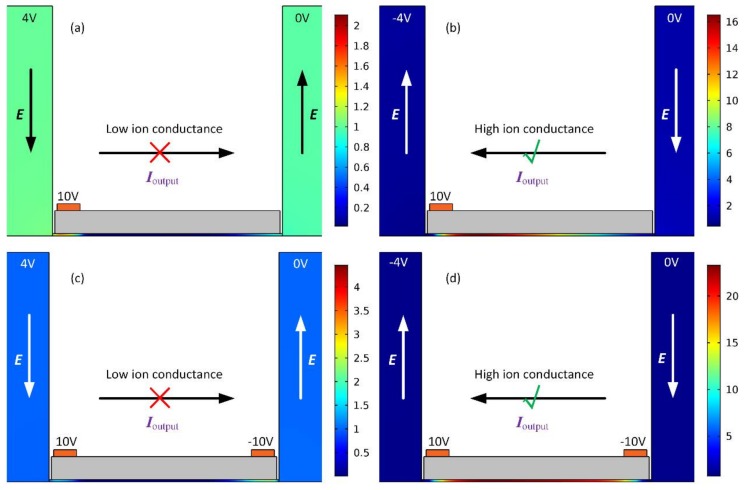
A surface plot of dimensionless ion concentration distribution (*C*_+_ + *C*_−_)/2*C*_0_ in two different electrode configurations for opposite working status of the proposed nanofluidic ion diode. (**a**,**b**) In the ionic device with single gate electrode mounted on the left of the top of the dielectric layer: (**a**) concentration field in the forward ‘off’ status in which the ionic current is blocked by the central desalinated area, and (**b**) the backward ‘on’ working state, in which the ionic current is enhanced by the central concentrated portion. (**c**,**d**) In stark contrast with the situation of merely one gate, the phenomenon of ion concentration polarization is made stronger and more stable in the structural design of ionic device with double gate electrodes laid on both sides of the dielectric layer surface; (**c**) as the applied field runs toward the D terminal, a low ion conductance at the channel center forbids any current flow in the forward direction, that is, the ‘off’ working state; and (**d**) with a reversal in the direction of imposed voltage gradient, the output current *I*_ouput_ toward the S terminal is intensified to great extent by the well-developed ion-enrichment zone inside the nanochannel.

The resulted ionic concentrations under the simultaneous action of vertical gating fields from the gate terminals and horizontal background electric field provided by the voltage difference across the S–D terminals are vividly shown in Figure 2. Under a positive voltage V_G1_ = 10 V imposed on the left gate terminal G1 and a negative voltage V_G2_ = −10 V applied to the right gate terminal G2, as anticipated from the schematics indicated by Figure 1, a background field ***E*** toward the downstream outlet port from a positive source voltage V_S_ = 4 V generates an ion-depletion zone at the midchannel in the non-electrode covered area (Figure 2a,c), due to the action of outward transport of induced counterions along the channel axial direction. The extremely high electrical impedance of the central ion-depleted zone ‘blocks’ any output ionic current *I*_output_ flowing towards the D terminal, i.e., the forward ‘off’ working state of the nanofluidic ion diode.

On the contrary, once the voltage polarity of S terminal is reversed, e.g., V_S_ = −4 V, an internal ion-enriched zone is established in the middle region (Figure 2b,d). This kind of enrichment in charge carriers reduces the system impedance and raises the output current flux *I*_output_ towards the S terminal to great extent, that is, the backward ‘on’ status of the ionic circuit platform.

**Figure 3:**
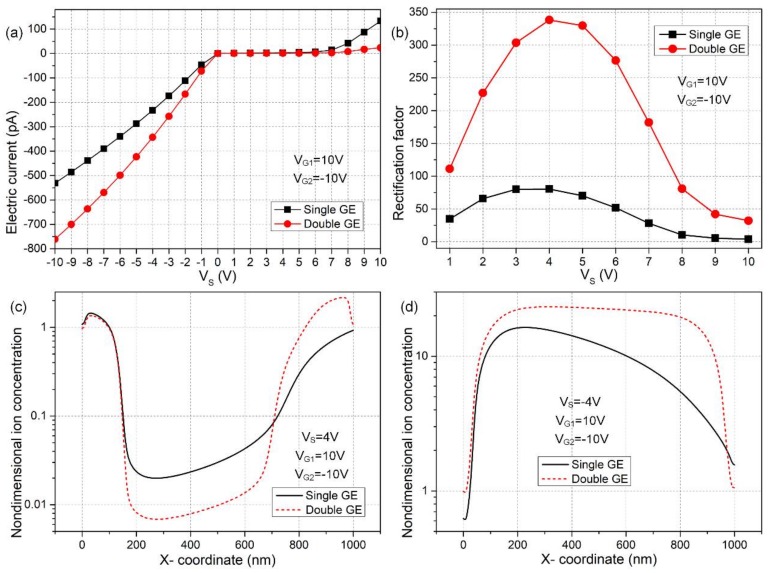
A quantitative comparison study between the rectification performance of the ionic device with single and double gate terminals for the given values of gate voltages V_G1_ = −V_G2_ = 10 V. (**a**) Output current flow *I*_output_ as a function of the source voltage Vs; (**b**) rectification factor of ionic current flux in reverse directions for the contrast between ‘on’ and ‘off’ status at varying magnitude of Vs; (**c**) nondimensional centerline concentration distribution of electrolyte charge carriers along nanochannel length direction for Vs = 4 V, in which the ionic species are sharply depleted in the central region, which appears to be more severe in the double GE design; (**d**) centerline ion density field for Vs = −4 V, in which a large amount of counterions are accumulated in the midchannel, and this ion-enrichment phenomenon takes place more appreciably for the advanced device design.

The specific rectification performance of ionic current for the nanochannel is calculated and quantified in Figure 3. In Figure 3a, the ionic current is severely inhibited in the forward direction while being appreciably enhanced in the backward direction, which is in qualitative accordance with the ion concentration distribution exhibited in Figure 2. Most importantly, the device configuration with double G terminals produces higher current flux for the ‘on’ working status and lower ionic current for the ‘off’ state than that with single gate electrode (Figure 3a), implying the nanofluidic ion diode with double gate electrodes is of a higher rectification performance in comparison to the situation of single G terminal.

The above deduction can be evidenced by the data plot of rectification factor as a function of the source voltage for the two distinct cases in Figure 3b. According to Figure 3b, the nano-scale ion diode is always much more efficient in performance when double G terminals are deposited on top of the dielectric layer. Besides, the rectification performance is a nonlinear function of the source voltage and reaches a peak value in the intermediate range around V_S_ = 4 V for both electrode configurations.

At a low value of Vs, the electrophoretic driving force of non-uniform surface conduction along the nanochannel is too weak at moderate Dukhin numbers, resulting in low device efficiency. Under the condition of a high source voltage, the interaction between V_S_ and V_G_ becomes important; the induce zeta potential is suppressed due to a small voltage difference between the S and G terminals, giving rise to an imperfect ion diode once again. As a result, the optimum device performance occurs at moderate source voltages. However, these simulation results suggest that we are allowed to apply a wide range of Vs, e.g., −8 V ≤ Vs ≤ 8 V for double G terminals, and a narrower range of Vs, e.g., −6 V ≤ Vs ≤ 6 V for single G terminal in practical experiment, and these particular ranges of Vs can be further expanded by employing larger gate voltages in both device configurations.

Subsequently, molar concentration distribution of electrolyte charge carriers is demonstrated by numerical calculation in Figure 3c,d, in order to explain the reasons for the higher rectification factor and wider working voltage range of the device design of double top gates (Figure 2c,d). From Figure 3c, under a positive source voltage V_S_ = 4 V, compared to the case of single G terminal, the middle desalinated region in the two-gates device not only occupies a larger scale within the nanochannel, but the related ionic concentration becomes much lower as well. That is to say, the dissipation of charge carriers is greatly aggravated in the presence of more gates, so that the ionic current is hindered more excessively in the forward direction. From Figure 3d, as for a negative voltage value of source terminal Vs = −4 V, both the scope and ion number density of the concentrated region is enhanced to great extent within the thin duct for the device design of double gates in comparison to the single gate configuration. Accordingly, the accumulation of ionic species is sharply boosted with two gates positioned on top of the insulation layer. In this way, the current flux is increased along the backward applied field with double G terminals compared to the situation of single gate due to an enhancement of the action from induced charge electrokinetic phenomenon.

So, the enhanced output current in the ‘on’ status and reduced ionic flux in the ‘off’ working state enable the device configuration of double G terminals (Figure 2a,b) to serve as a more advanced design of on-chip nanofluidic ion diode than that using merely one top gate electrode. This new micro/nanofluidic hybrid system possesses enormous potential to accelerate the pace of progress for forthcoming liquid-phase-based intelligent integrated circuits.

### 3.2. Effect of Gate Voltage Magnitude

**Figure 4:**
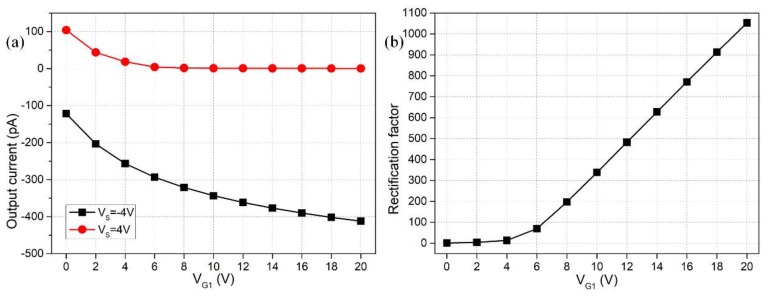
Simulation analysis of the influence of gate voltage magnitude on the device performance of the double-gate nanofluidic ion diode: (**a**) output ionic current as a function of gate voltage and (**b**) V_G_-dependent rectification factor for a source voltage contrast between V_S_ = 4 V and V_S_ = −4 V.

Having demonstrated the superiority of double GE device design in Section 3.1, it is then necessary to make it clear how the DC voltages applied to the two gate terminals influence the rectifying function of the nanofluidic ion diode, while the source voltage for reverse working states is given, i.e., Vs = ±4 V (Figure 4). As shown in Figure 4a, with increasing V_G1_ from 0 V to 20 V accompanied by a decrease of V_G2_ from 0 V to −20 V, the forward current flow for the ‘off’ state is more seriously refrained and almost approaches zero as the gate voltage magnitude surpasses 14 V, and the backward ionic current for the ‘on’ state correspondingly grows up to −380 pA under the same V_G1_ and V_G2_. The rectification factor of our ionic device as a function of V_G_ is quantified in Figure 4b, and the V_G_-dependent device performance follows obviously a nonlinear growth trend, i.e., $\gamma =V_{G}{}^{\beta }$ with *β* > 1, in that the gradient of the curve becomes larger with increasing V_G_. This indicates that electrochemical transport of non-uniform-induced counterions through both electrophoretic and electroconvective charge transfer is responsible for the working principle of the proposed nanofluidic ion diode (nonlinear electrokinetics), rather than the homogenous natural Debye screening charge compensating the fixed surface charge density on nanochannel sidewalls (the linear electrokinetics). The above simulation analysis sheds light on the importance of applied gate voltages to the two G terminals in reinforcing induced-charge electrokinetic phenomenon inside the nanochannel, an increase which is able to induce greater internal depleted/enriched concentration polarization for the two opposite working states, respectively, and enhance the resulted rectification factor of the ionic circuit platform as well.

It is noteworthy that although an enhancement in gate voltage magnitude is beneficial for improving the rectification performance, severe electrochemical reactions from electron phase transfer would be activated on the Stern layer surface under an excessively large V_G_, the phenomenon of which is not included in current mathematical model. The electrode reactions can cause fatal damage to the gate electrodes by inducing galvanic corrosions. Accordingly, we slightly raised the gate voltage magnitude from V_G1_ = −V_G2_ = 10 V to V_G1_ = −V_G2_ = 12 V in subsequent analysis to avoid these detrimental effects at large Dukhin numbers.

### 3.3. Effect of Native Surface Charge Density on Nanochannel Sidewalls

According to preceding analysis, the ionic diode works on the basis of non-uniform-induced counterions due to polarization of the dielectric coating under vertical gating electric fields. However, the spontaneous Debye screening from the fixed surface charge density next to the electrolyte/insulating layer interface generates a uniform distribution of ionic charges, which imposes a negative impact on the inhomogeneity of internal charge carriers required for the nanodevice to work with high efficiency. From Equation (15), the growth of fixed surface free charge density poses a higher demand on the applied gate voltages for electrical manipulation of the nanoscale diode. So, for given voltage values of both G terminals, the device performance would be lowered with an increase in the magnitude of *σ_free_* due to enhanced linear electrokinetics in front of induced-charge electrokinetics.

**Figure 5:**
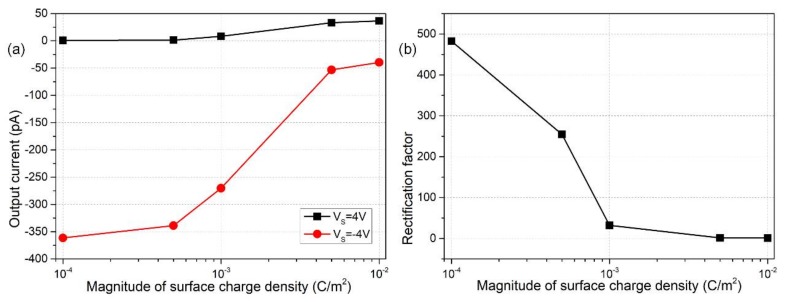
V_G1_ = V_G2_ = −12 V: influence of surface charge density distribution on diode functionality of the nanofluidic device. (**a**) Output current flux as a function of *σ_free_* in reversed working status of the nano-dimensional device for V_S_ = 4 V and V_S_ = −4 V and (**b**) *σ_free_*-dependent rectification performance of the ionic diode.

We can witness above conjectures by having a look at the specific computational data in Figure 5. From Figure 5a, the output current for the forward ‘off’ status unexpectedly increases, and that for the backward ‘on’ status sharply decreases as the fixed interfacial charge rises in magnitude. As a consequence, the rectification factor diminishes at higher surface charge densities, and the nanofluidic device even loses the diode functionality when *σ_free_* approaches −0.01 C/m^2^ as shown in Figure 5b. Supported by above results from direct numerical simulation, an enhancement in fixed surface charge tends to homogenize the internal distribution of charge carriers by relatively suppressing the induced counterions from nonlinear electrokinetics, resulting in milder concentration polarization that leads to a reduced rectification performance.

### 3.4. Effect of Electrolyte Concentration

**Figure 6:**
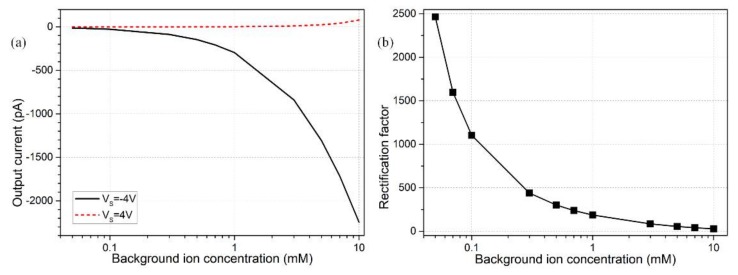
Effect of buffer ionic conductivity on the field-effect current control at nanometer dimension: (**a**) ion concentration-dependent *I*_output_ for reversed device working states; (**b**) rectification factor as a function of the medium conductivity.

The free surface charge density is then increased to −0.001 C/m^2^ so it is closer to actual experimental conditions. It is the enhancement in linear electrochemical polarization that asks us to raise the gate voltage magnitude to higher values, e.g., V_G1_ = −V_G2_ = 15 V for engendering stronger nonlinear electrokinetics with greater induced bipolar counterions under the electrode covering area. However, this can effectively generate the desirable ionic diode functionality for electrolyte concentrations less than 1mM (Figure 6b). Nevertheless, even if in the presence of larger gate voltages, the device performance decays and even becomes negligibly small for ionic conductivity exceeding *σ_f_* = 0.0153 S/m (*C*_0_ = 1 mM) due to the weakened surface conductivity when the Debye screening length cannot run throughout the nanochannel height direction.

Since the Ohmic current is linearly proportional to the liquid conductivity, the forward current increases with electrolyte concentration, albeit ion enrichment is more severely refrained with a shrink of the double-layer thickness (black line in Figure 6a). At the same time, the ion-depletion zone cannot be well developed at the channel central part as well for the backward ‘off’ status, rendering a quicker growth rate of output current flux with increasing ionic conductivity (red line on Figure 6a). As a consequence, as long as the double-layer thickness is no more than the vertical size of nanochannel, the device efficiency can be greatly reduced, since it is the non-uniform surface conduction of induced counterions within the diffuse screening cloud that initiates internal concentration polarization, which fails to occur without a healthy double layer extension inside the nano-duct.

In this sense, since the Debye length shrinks with an increase in liquid conductivity, for a given channel height H_N_, there is invariably a threshold ion density, only below which the nanofluidic device can realize ionic current rectification and diode functionality with complete extension of the induced double-layer. Besides, the critical background ion concentration is inversely proportional to the squared value of H_N_ from Equation (17). So, as a higher ionic conductivity passes through the perm-selective nanoporous medium, the nanochannel height has be made lower in the microfabrication process to match the smaller Debye layer extension in order for the normal ionic circuit platform to operate. That is, the usage of higher conductivity liquid medium poses a higher demand on the characterized size of the ion-exchange medium, e.g., for a background concentration *C*_0_ = 10 mM, the lateral dimension of the nanofluidic channel ought to be no more than 3.05 nm, which equals the Debye screening length for electrolyte conductivity of 0.153 S/m. Accordingly, for biological buffer medium with ionic concentration approaching 100 mM (*σ_f_* ≈ 1.53 S/m), the nanochannel height is required to be lower than 1nm for causing effective field-effect current control using current nanodevice.

### 3.5. Effect of the Dielectric Layer Thickness

As analyzed above, the double layer extension along the nanochannel transversal direction plays a very important role in inducing the non-uniform surface conductivity for field-effect current control of the ion diode. The background ionic conductivity is set to *C*_0_ = 1 mM with a double layer thickness of *λ* > 9.6 nm, so that it is still suitable for establishing the nanochannel with a height of H_N_ = 10 nm. The surface charge density −0.001 C/m^2^ remains unchanged in later discussions; so does the gate voltage magnitude of V_G1_ = −V_G2_ = 15 V and source voltage of Vs = ±4 V.

From Equations (14) and (15), both electrical polarizability and geometrical size of the dielectric layer exert a significant influence on induced charge electrokinetics within the nanochannel. Since the insulation layer is regarded in series connection with the double layer capacitance, a lower height of dielectric coating would make less voltage fall within itself, therefore producing stronger non-uniform zeta potential and greater induced counterions along the thin duct, which would bring about tremendous benefit to actuating ICP interface at the midchannel for achieving field-effect current control. 

**Figure 7:**
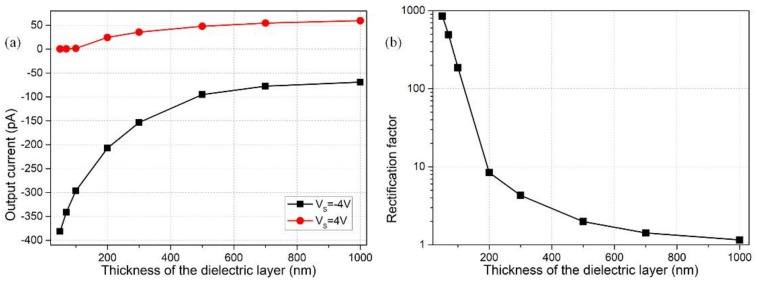
Simulation analysis of the effect of dielectric layer thickness on nanodevice performance for voltage combinations of V_S_ = ±4 V and V_G1_ = −V_G2_ = 15 V: (**a**) thickness-dependent output ionic current for both ‘on’ and ‘off’ working states; (**b**) rectification factor as a function of the thickness of insulating coating.

These qualitative analyses are quantitatively proved by the simulation results shown in Figure 7. According to Figure 7a, an increase in the thickness of insulation coating evidently suppresses the output current in the backward ‘on’ status and enhances the ionic flux in the forward ‘off’ status for the nanofluidic ion diode, which is adverse for ionic current rectification in the two gates configuration (Figure 7b). As emphasized previously, the reason behind this is the diminishment of induced polarization of the dielectric layer with increasing thickness, giving rise to fewer counterions from induced-charge electrokinetics, so that ion enrichment/depletion at the center of internal nanochannel is greatly weakened with a thick dielectric layer for the on/off status, respectively. As a consequence, in practical experiments, it is better to construct a covering layer as thin as possible, so as to realize ideal field-effect ionic-current control.

### 3.6. Influence of Dielectric Permittivity of the Insulation Covering Layer

**Figure 8:**
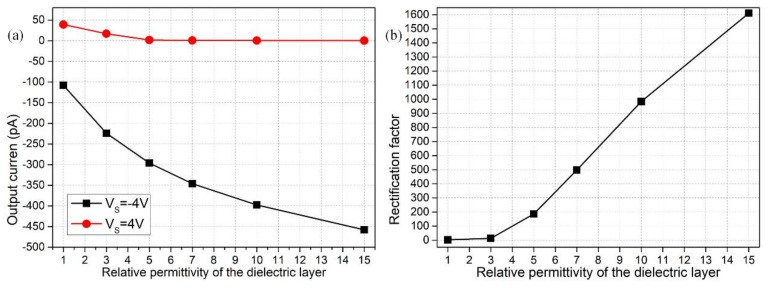
Effect of dielectric permittivity of the thin insulation layer on rectification performance of the nanofluidic ion diode: (**a**) permittivity-dependent output ionic current for both ‘on’ and ‘off’ working states; (**b**) rectification factor as a function of relative permittivity of the dielectric coating.

The insulation covering layer not only profoundly affects the diode functionality in terms of its lateral dimension, but the importance of its dielectric permittivity in the device performance cannot be overlooked as well. From Equation (15), the threshold gate voltage for generating effective field-field current control is decreased as dielectric layer permittivity increases. A plausible explanation for this phenomenon can be found in Equation (14b); in analogy to the reduction of the insulating layer thickness, an enhanced dielectric permittivity of the thin coating would also improve its electrical polarizability, thereby inducing more inhomogeneous counterions in the presence of vertical gating fields on both sides. This identically reinforces internal concentration polarization for reversed working status (resulting in increased output current for the ‘on’ state indicated by the black line, and decreased ionic flux for the ‘off’ state indicated by the red line in Figure 8a with dielectric permittivity) and subsequently boosts the rectification factor for the nanofluidic ion diode as shown in Figure 8b.

### 3.7. Effect of Geometrical Configuration of Double Gate Terminals

Dielectric permittivity of the dielectric membrane is deliberately enhanced to a higher value $\varepsilon_{ins}=10\varepsilon_{0}$, while its thickness is kept constantly at H_ins_ = 100 nm to slightly enhance the diode functionality. On having clarified the importance of dielectric layer property in nano-scale field-effect current control, we then focus on how the discrete arrangement of the double gate terminals affects the device performance.

#### 3.7.1. Effect of GE Width

**Figure 9:**
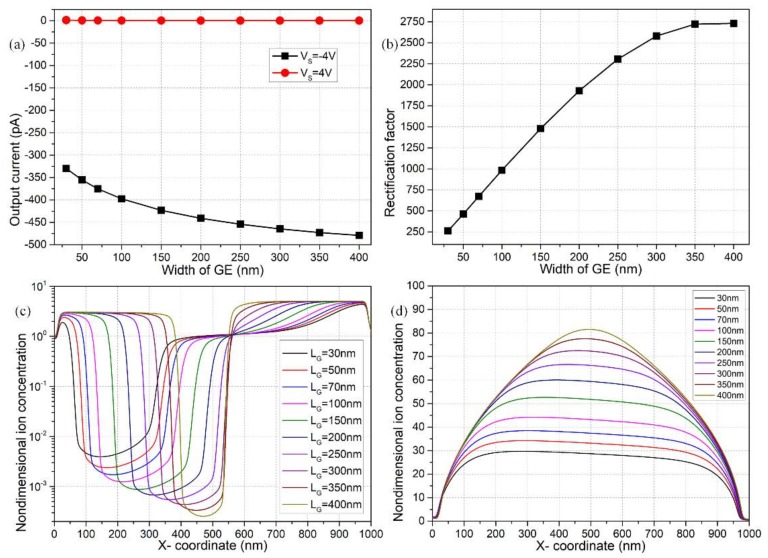
On the effect of GE size on the device performance: (**a**) *I*_output_ for reversed working states of the nanofluidic ion diode; (**b**) rectification factor as a function of GE width; (**c**) internal depleted ion concentration distribution for the forward ‘off’ status with V_S_ = 4 V; (**d**) internal enriched distribution of charge carriers for backward ‘on’ status with V_S_ = −4 V.

The analysis in this section is directly based on the calculation results shown in Figure 9. According to Figure 9a, an expanded size of the two gate electrodes can inhibit/enhance the forward/backward output current for the off/on working states, respectively, resulting in improved ionic current rectification of the fluidic device (Figure 9b). This is caused by a raise in the number density of dipolar induced counterions under a larger covered area of GE; therefore, the non-uniform surface conduction that is enhanced can give rise to greater concentration polarization at the midchannel, as shown in Figure 9c,d.

On one hand, under the ‘off’ status with V_S_ = 4 V, as the width of double GE is increased gradually from 30 nm to 400 nm, the area occupied by the bipolar-induced counterions is enlarged to cause more severe internal ion depletion, so that the concentration of charge carriers goes down further at the channel center (Figure 9c). At the same time, the ion concentration distribution becomes much more symmetrical with respect to the channel centerline, which may help suppress the forward current from another aspect. On the other hand, in the ‘on’ working state with V_S_ = −4 V, the midchannel undertakes more enhanced ion enrichment in the presence of wider gate electrodes, since the induced counterions take up a larger region on both sides that causes more effective inward transport of charge carriers to engender stronger enriched concentration-polarization. These results imply we can take advantage of broader gate terminals to engender better ionic current rectification and diode functionality, due to an enhanced concentration polarization from bipolar induced-charge electrokinetics.

#### 3.7.2. Effect of GE Position

Although enlarging the size of GE can effectively improve the device performance, there is an inferior limitation in the inter-electrode separation due to an increased difficulty in microfabrication process of a smaller gap size. Accordingly, we select a reasonable value of electrode width L_G_ = 200 nm in the intermediate range. It is then necessary to seek for other methods to modify the discrete electrode configuration for enhancing the diode functionality.

**Figure 10:**
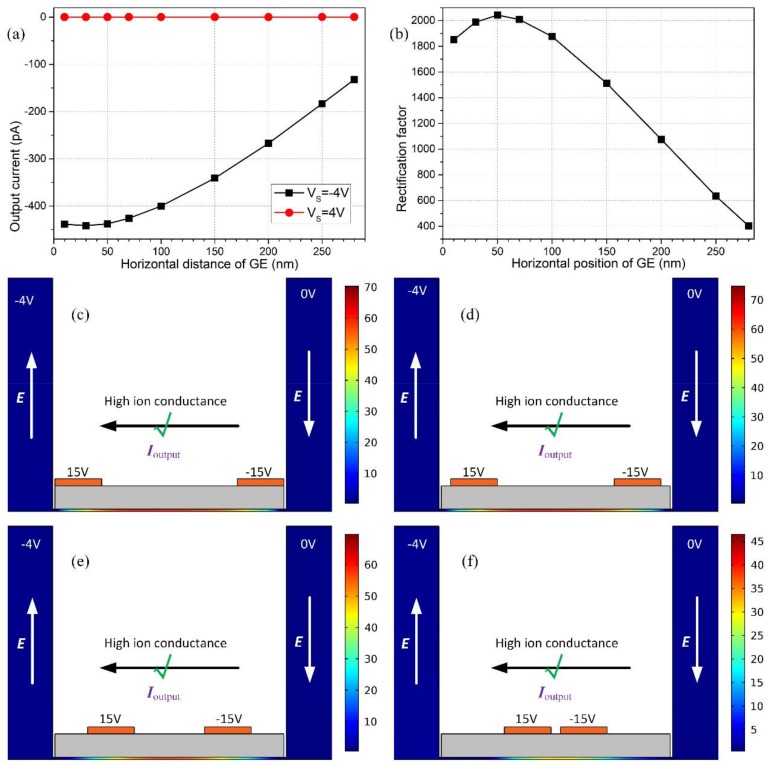
Effect of the location of double gate terminals on the resulted nanodevice performance. (**a**) The output current flux under reversed working states at V_S_ = ±4 V; (**b**) rectification factor as a function of the horizontal position of GE (L_D_). (**c**–**f**) A surface plot of dimensionless concentration distribution of charge carriers for the backward ‘on’ status at Vs = −4V with different electrode positions, at (**c**) L_D_ = 10 nm, (**d**) L_D_ = 50 nm, (**e**) L_D_ = 150 nm, and (f) L_D_ = 280 nm.

In fact, the specific location of electrodes also plays a significant role in amending the rectification performance. As shown in Figure 10, as the electrode location L_D_ (the nearest distance between one side of dielectric layer and adjacent gate electrode as shown in Figure 1a) shifts closer to the center of insulation coating surface (Figure 10c–f)), the area within which ion concentration polarization takes place is more severely restricted, so that fewer charge carriers are enriched under the non-covered area of double gate terminals for a negative *V_S_*, which forbids the backward ionic current flow to greater extent (Figure 10a) and results in a reduced rectification factor at larger L_D_ (Figure 10b). This conclusion suggests that there is no need for us to arbitrarily deploy the pair of gate electrodes in the immediate vicinity of either the edge or the central area of the thin dielectric layer on top of the nanochannel, relaxing greatly the fabrication procedure in practice.

### 3.8. Analysis of Electroosmotic Streaming

**Figure 11:**
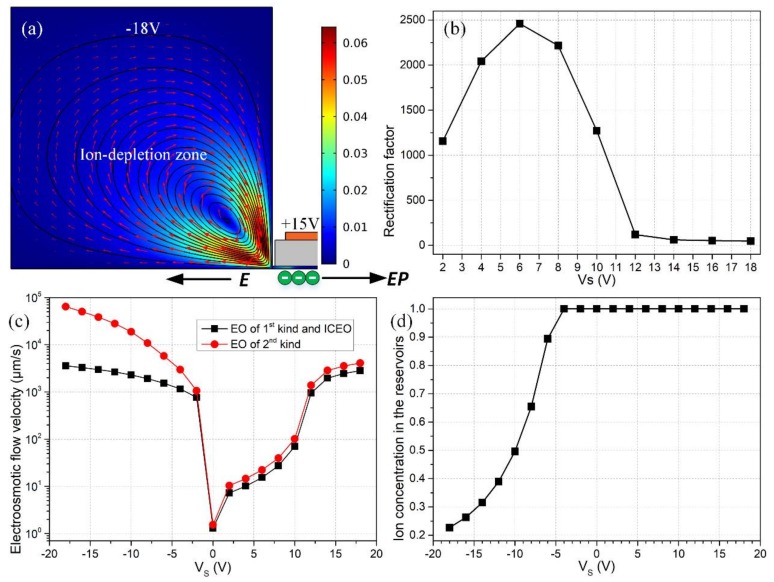
A simulation analysis of the effect of different kinds of electroosmotic flow and ion concentration polarization in the micrometer-scale reservoirs on ionic current rectification of the nanofluidic ion diode. (**a**) For V_S_ = −18 V, V_G1_ = −V_G2_ = 15 V, the ion-depletion zones induced in microchambers due to mismatch of charge carriers at the micro/nanofluidic interfaces arouse vortex flow field of 2nd electroosmosis with oppositely rotating directions on both sides, in which only the left reservoir is presented with its counterpart on right side not shown; (**b**) rectification performance of the nanodevice as a function of V_S_; (**c**) quantification on electroconvective flow velocity of 1st EOF and ICEO inside the nanochannel, as well as 2nd EOF in reservoirs on both sides as a function of V_S_; (**d**) V_S_-dependent characteristic ion density in the microchambers (concentrations of charge carriers are identical in reservoirs on both sides).

Although a variety of electroosmotic flow has been introduced briefly in Section 2.1, it is still necessary for us to conduct quantificational analyses on the physics of fluids appearing in the nano-scale ion-circuit platform. As shown in Figure 11a, for a negative value of V_S_ = −18 V, a large amount of counterions induced by external gate terminals quickly transport to the midchannel, so that the ionic species are severely depleted in both reservoirs. At a sufficiently large applied electric field along the channel length direction, the ion depletion zones can be well developed on both sides, and 2nd EOF due to the Coulomb force within the extended space charge layer occurs in vortex-flow patterns in the over-limiting current region (Figure 11a). As shown in Figure 1b,c, ICEO and 1st EOF can work synergistically with electrophoretic transport of ionic species, all of which are in favor of convective charge transfer towards the desired directions for the two distinct working states. This can account for why the device performance increases with V_S_, as the absolute value of source voltage is no more than 6 V.

However, as V_S_ is less than −6 V, whirlpools of 2nd EOF in ion-depleted reservoirs (Figure 11d) begin to dominate over ICEO within the nanochannel (Figure 11c). In the meantime, for $\left|V_{S}\right|\geq 6V$, the device rectification performance drops rapidly with increasing magnitude of source voltage. In this sense, due to the existence of such a coincidence, it should be the electroconvection from 2nd EOF in backward ‘on’ status that lowers down the device efficiency.

In fact, 2nd EOF in the over-limiting current region can enhance the electrical current in the microchambers to some extent by inducing strong convective charge transfer, in comparison to limiting current region without a full-developed space charge layer. This indirectly disturbs the ion concentration distribution in the reservoirs on both sides and increases the in-situ ion densities. To maintain the global mass conservation, however, the concentration of electrolyte charge carriers has to be restrained in the central ion-enrichment region for V_S_ ≤ −6 V. Therefore, the backward output current for the ‘on’ status is unexpectedly reduced due to the actuation of 2nd EOF in adjacent microchambers, so that the rectification factor, which is linearly proportional to the current flux in the ‘on’ working state with a negative V_S_, is suppressed as well for $\left|V_{S}\right|\geq 6V$.

**Figure 12:**
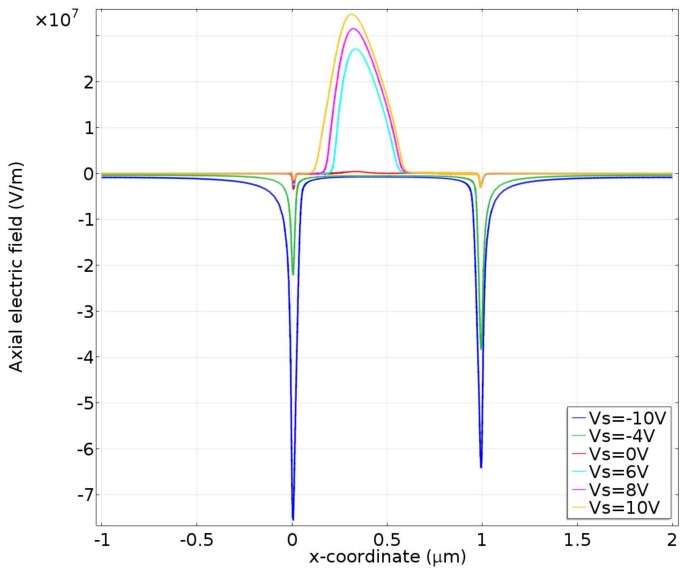
Distribution of axial electric field along the nanochannel centerline for different values of the source voltage V_S_.

More importantly, the action of the applied electric field on the induced counterions initiates ICP, which makes a response and in turn changes the distribution of local axial field. For instance, with a positive bias of Vs, the central depleted region witnesses a higher field intensity than the enriched regions on both sides considering current conservation, and vice versa with the negative counterpart (Figure 12). This feedback implies that a high enough applied potential for such a short channel could possibly overcome or at least weaken the concentration polarization effects that lead to the diode behavior. Figure 12 exhibits the axial electric field on the centerline for varying source voltages, indicating that (1) the EDL potential gradient at the inlet is pretty much eliminated for higher positive applied source voltages and (2) the gradient at the outlet is also weakened for higher voltages, which weakens the overall ICP and subsequent ion rectification. So, the situation seems more sophisticated than just the electroconvection from EOF of 2nd kind at the micro/nanofluidic interfaces. This was previously explained in Ref. [51] in the context of the competition between the applied field and intrinsic field and how one can lower the outlet potential gradient relative to the applied field (or increase the applied field relative to the EDL gradient, in their case) to actually allow negative co-ions into the channel to accumulate even in the “off” mode—which would definitely decrease the rectification effect.

## 4. Conclusions

In summary, by reinforcing internal concentration polarization of a nanofluidic channel connected to microchambers on both sides through the induced-charge electrokinetic phenomenon at the nanometer dimension, we have proposed a unique structural design of the field-effect-tunable nanofluidic ion diode with double gate terminals laid on top of a thin dielectric covering layer. According to the simulation analyses and discussions on the effect of various geometrical and physicochemical parameters, the capability of field-effect ionic-current control has close bearings on the non-uniform surface conduction of induced bipolar counterions within the EDL, which can well extend inside the nanochannel for moderate medium conductivities. A rectification factor as high as one thousand-fold can be realized within a wide range of parametric space, whereas attention should be paid to the adverse influence of 2nd EOF in the backward ‘on’ working state. The geometric size of the current nanofluidic device is easily scalable; for example, the length of nanochannel, the thickness of dielectric layer, the span of double gate electrodes, and the size of microchambers on both sides can get either larger or smaller at our wishes, and the resulted diode functionality would not be severely affected as long as sufficiently large gate voltages were imposed on the double gate terminals, which makes the nanofluidic chip an eligible building brick for the small-scale, on-chip ionic circuit platform. Supported by mathematical analysis, our demonstration makes it possible to better take advantage of the dual-gates-based micro/nanofluidic system to developi a high-efficiency nanofluidic ion diode for forthcoming liquid-phase integrated circuits.
